# Heavy metal contamination in pristine environments: lessons from the Juan Fernandez fur seal (*Arctocephalus philippii philippii*)

**DOI:** 10.1098/rsos.221237

**Published:** 2023-03-29

**Authors:** Constanza Toro-Valdivieso, Ravin Jugdaohsingh, Jonathan J. Powell, Joseph I. Hoffman, Jaume Forcada, Charles Moore, Barbara Blacklaws

**Affiliations:** ^1^ Department of Veterinary Medicine, University of Cambridge, Madingley Rd, Cambridge CB3 0ES, UK; ^2^ Biominerals Research Laboratory, Department of Veterinary Medicine, University of Cambridge, Madingley Rd, Cambridge CB3 0ES, UK; ^3^ British Antarctic Survey, High Cross, Madingley Rd, Cambridge CB3 0ET, UK; ^4^ Department of Animal Behaviour, Bielefeld University, Bielefeld 33501, Germany; ^5^ Algalita Marine Research Foundation, 148N Marina Dr, Long Beach, CA 90803, USA

**Keywords:** cadmium, mercury, silicon, heavy metals, marine mammal, pinniped

## Abstract

Heavy metals, including mercury (Hg) and cadmium (Cd), occur naturally or anthropogenically and are considered toxic to the environment and human health. However, studies on heavy metal contamination focus on locations close to industrialized settlements, while isolated environments with little human activity are often ignored due to perceived low risk. This study reports heavy metal exposure in Juan Fernandez fur seals (JFFS), a marine mammal endemic to an isolated and relatively pristine archipelago off the coast of Chile. We found exceptionally high concentrations of Cd and Hg in JFFS faeces. Indeed, they are among the highest reported for any mammalian species. Following analysis of their prey, we concluded that diet is the most likely source of Cd contamination in JFFS. Furthermore, Cd appears to be absorbed and incorporated into JFFS bones. However, it was not associated with mineral changes observed in other species, suggesting Cd tolerance/adaptations in JFFS bones. The high levels of silicon found in JFFS bones may counteract the effects of Cd. These findings are relevant to biomedical research, food security and the treatment of heavy metal contamination. It also contributes to understanding the ecological role of JFFS and highlights the need for surveillance of apparently pristine environments.

## 1. Introduction

Marine pollution is a matter of global concern. Furthermore, the negative effects of pollutants (e.g. heavy metals, microplastics and persistent organic pollutants) on marine environments and the species that rely on them, including humans, have been widely assessed and reviewed [1,2]. In marine environments, the anthropogenic release of toxic compounds can spread far from the original source of contamination through ocean currents, atmospheric dispersion and vectors including plastic debris [2–4].

Heavy metals are metal or metalloid elements of high atomic mass. Heavy metals such as cadmium (Cd) and mercury (Hg) are not known to have any biological or essential functions in mammals. On the contrary, they are of special concern due to their high toxic potential [5]. However, in marine environments, Cd is considered a micronutrient for some phytoplankton inhabiting oligotrophic environments such as the South Pacific subtropical gyre [6,7]. Unlike man-made compounds, these elements have natural sources. However, human activities such as mining and oil extraction have been linked to an increased release of toxic heavy metals into marine environments [3,8,9]. According to the Agency for Toxic Substances and Disease Registry (ATSDR), Hg and Cd are among the most hazardous [5]. In the case of Hg, toxicity and bioavailability varies depending on its form: metallic element, inorganic salts or organic compounds. Methylmercury (MeHg), the most toxic form, is known to cause microtubule and mitochondrial destruction, increased lipid peroxidation and accumulation of neurotoxic molecules [10]. Cd, on the other hand, affects gene expression, inhibits DNA repair, interferes with apoptosis and autophagy, induces oxidative stress and interacts with bioelements such as calcium [11–13].

Marine mammals are known for their capacity to bioaccumulate and biomagnify contaminants, and there is evidence showing these species have evolved various adaptation repertoires that make them more resilient to heavy metal contamination [14]. For instance, marine mammals have high numbers of metallothioneins in relevant organs such as the liver and kidney to detoxify heavy metals [15]. However, despite evolving decontamination mechanisms, marine mammals may still suffer from heavy metal toxicity [16,17], and the threshold of a marine mammal's resilience to heavy metals is not well known. Indeed, a review on the immunotoxic effects of environmental pollutants in marine mammals published by Desforges *et al*. [18] suggested that the concentration (ppm) of Hg, MeHg and Cd required to adversely affect lymphocyte proliferation varied among marine mammal species (0.002–1.3, 0.009–0.06 and 0.1–2.4 ppm respectively) [18].

To the best of our knowledge the Juan Fernandez fur seal (JFFS) has received only sporadic and intermittent monitoring in the last two decades. The JFFS (*Arctocephalus philippii philippii*) is a marine mammal endemic to the Juan Fernandez archipelago (JFA) and the Desventuradas Islands, with breeding colonies on the former. This remote and semi-pristine location is considered a hotspot of biodiversity and endemism [19]. Between the late eighteenth and early nineteenth centuries, the species became an important target for the pelt industry. Furthermore, it is estimated that almost four million JFFS skins were harvested during the sealing period [20]. Due to overexploitation, the species was presumed extinct from the late ninteenth century until the early 1960s [21]. It was not until 1995 that the Chilean government gave the species complete legal protection for a period of 30 years. Since its rediscovery, the species has shown a steady population recovery, from a couple of hundreds to an estimated hundred thousand individuals [21–24]. Although this increase is positive news, the numbers remain well below the pre-sealing records. This limited population size and narrow geographic range mean the species' survival remains vulnerable to catastrophic events. The International Union for Conservation of Nature (IUCN) has advised further research and close monitoring [25].

As part of an extensive study on this species, we evaluated heavy metal exposure in JFFS using inductively coupled plasma mass spectrometry (ICP). More specifically, our aims were (i) to identify common heavy metals JFFS are exposed to and their prevalence, and (ii) to identify possible sources of exposure. Cd exposure was highest in JFFS and so the final part of the study focused on the effects of Cd in JFFS to answer the following questions: is Cd bioavailable and being absorbed and incorporated into JFFS bones? If this is the case, what is the impact of Cd on their bone structure?

## 2. Methods

### 2.1. Sample collection

#### 2.1.1. Faeces

**Figure 1.**
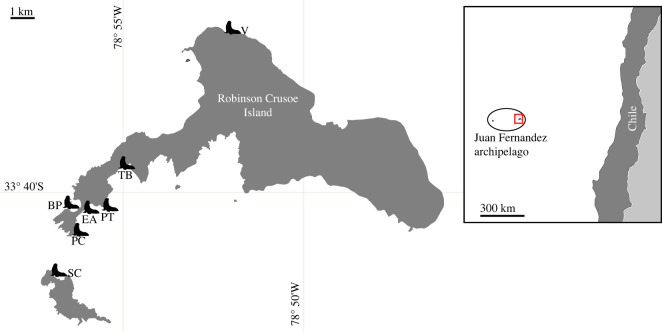
Simplified map of Robinson Crusoe and Santa Clara islands in the Juan Fernandez archipelago. Fur seal icons show the sampling locations. Bahia el Padre (BP), Santa Clara (SC) and Tierras Blancas (TB). Figure modified from [26].

This research took place in the Juan Fernandez archipelago (JFA). Fourteen faecal samples were collected from three different JFFS colonies; Bahia el Padre (BP, *N* = 4), Tierras Blancas (TB, *N* = 5) and Santa Clara (SC, *N* = 5), covering two of the three islands that make up the archipelago as shown in figure 1. After collection, faecal samples were placed in Nalgene 5005-0015 Specimen Cryogenic Vials. As the faeces were collected opportunistically, host information such as sex or age was not available. Samples were stored at −20°C within 32 h of collection and stored for 1–2 months until transfer to the laboratory, where they were stored at −30°C until analysis. All JFFS faecal samples were collected with the logistic support of CONAF during the 2017–2018 reproductive season.

Additionally, five Antarctic fur seal (*Arctocephalus gazella*, AFS) faecal samples were collected from Bird Island, South Georgia, Antarctica (54°00′ S, 380°3′ W) also during the 2017–2018 reproductive season as part of a long-term monitoring programme conducted by the British Antarctic Survey (BAS).

#### 2.1.2. Bone samples

To examine heavy metal concentrations in JFFS bones, 10 bone fragments were opportunistically collected during the 2018–2019 reproductive season. All the bones were lower mandibles from adults (*n* = 5) and pups (*n* = 5) of undetermined sex or exact age.

For comparison, two archived bones samples were included in this analysis. Both samples were made up of skull fragments. One of the samples belonged to an adult male grey seal (*Halichoerus grypus atlantica*, GS) collected in the Orkney Islands, Scotland, in 2003. The other sample corresponded to an adult male Antarctic fur seal collected from Bird Island, South Georgia, in 2019.

#### 2.1.3. Non-pinniped samples

Although little is known about the JFFS diet and foraging behaviour, pelagic species including myctophid fish and squid have been identified as predominant components of their diet [27]. Additionally, predation of octopus inhabiting the shallower waters adjoining the JFA coast is often observed by the local community. In this study, we had access to some prey samples which we analysed to explore possible sources of heavy metals in the fur seal food chain. Eleven myctophid fish samples were opportunistically collected during a microplastic monitoring survey across the South Pacific subtropical gyre. Additionally, internal organs of three octopuses were donated by members of the local community.

Finally and because of the collection method, we expected some degree of contamination of the faecal samples with the surrounding soil, even though we tried our best to limit soil contamination while sampling. This contamination was likely to influence the concentration of some elements in JFFS faeces. Therefore, trace element levels in soil and water samples from the areas where JFFS faecal and bone samples were collected were also measured to allow sample contamination by the environment to be considered. Soil samples for the corresponding AFS faecal samples were not available for trace element and heavy metal analysis.

### 2.2. Trace element analysis

First, all faecal samples were air dried in a clean class II microbiological safety cabinet to eliminate any variation in water content between samples. Aliquots (0.1 g) of the dried samples were then digested with ultra-pure (UHP) nitric acid and hydrogen peroxide at room temperature, and the digest diluted with UHP water. Sample blanks were similarly prepared. The diluted samples and blanks were then measured for the concentrations of 53 different elements with a triple quadrupole inductively coupled plasma mass spectrometer (8900 ICP-MS/MS, Agilent Technologies Inc, CA, USA), using appropriately prepared multi-element calibration standards. In addition to calibration standards and appropriate blanks, a multi-element standard reference material (NIST Ref 1643) was run alongside each batch of samples; at the start, middle and end of the analysis run. The reference material contained 6.4 µg kg^−1^ cadmium and recovery was 100% on all occasions. Food chain samples were similarly prepared for ICP-MS/MS analysis, except the samples were not dried prior to acid digestion. Water samples were diluted in 1% UHP nitric acid prior to analysis, while soil samples were digested in an acid digestion microwave (UltraWave, Milestone SRL), diluted and analysed with the ICP-MS/MS. Cd and Hg concentrations in water samples were 0.0 ng g^−1^ or below the detection limits. Bone samples were first thoroughly cleaned of external surface contaminants by sonicating several times in ultra-pure water in an ultrasonic bath. Cleaned bones were then dried and aliquots digested in the acid digestion microwave. The acid digests were diluted in UHP water and analysed with the ICP-MS/MS. Cadmium and silicon levels in the faecal and bone digests by ICP-MS/MS, were confirmed with the analysis repeated on an ICP-optical emission spectrometer (Ultima 2C ICP-OES; Horiba Jobin-Yvon). Sample digestions and ICP-MS/MS and ICP-OES analyses were carried out by the biomedical research group at the Department of Veterinary Medicine, University of Cambridge.

### 2.3. Statistical analysis

All statistical analyses were performed in R v. 4.1.0 [28]. The code used in this study can be accessed in https://github.com/Cotissima/JFFS_HeavyMetal_analysis. First, we focused on the faecal and soil samples. The risk of analysing over-represented elements due to soil contamination was limited by performing a principal component analysis (PCA) and a cluster analysis to identify elements present at high concentrations in the faecal samples but low in the soil samples. Lower concentrations of these elements in soil samples meant their high concentrations in the faecal samples were unlikely to be the result of cross-contamination with soil (at the time of collection).

Next, we used Spearman rank correlation to look for possible statistical relationships between the selected elements. Finally, and due to the uneven sample sizes, we performed non-parametric Mann–Whitney *U*-tests at the 0.05 significance level to compare the concentration of the chosen elements between fur seal species.

The same approach as the one used for the faecal samples was applied to JFFS bones samples. PCA and cluster analysis were performed to identify possible over-represented elements due to cross-contamination with soil, followed by a non-parametric Mann–Whitney *U*-test at the 0.05 significance level to compare the elements concentrations between age groups.

## 3. Results

### 3.1. Trace element analysis of faecal samples

**Figure 2.**
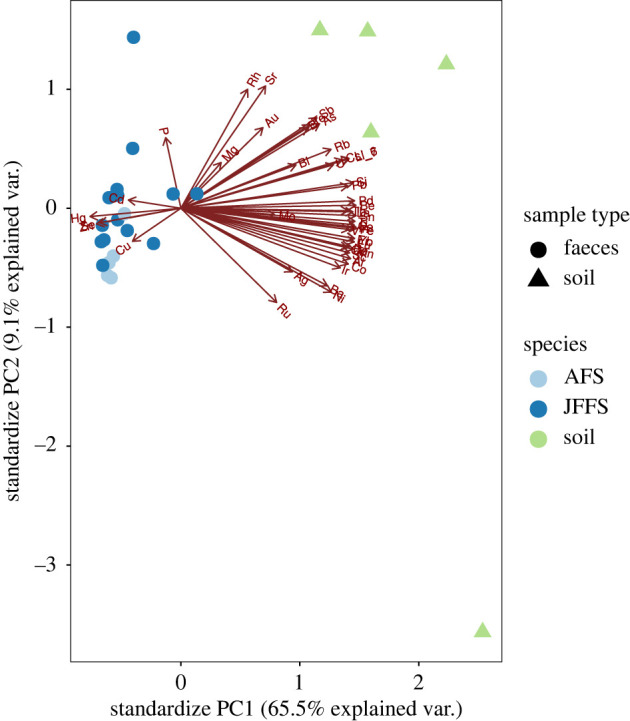
Principal component analysis (PCA) of the relationship between trace elements found in faecal and soil samples.

**Figure 3.**
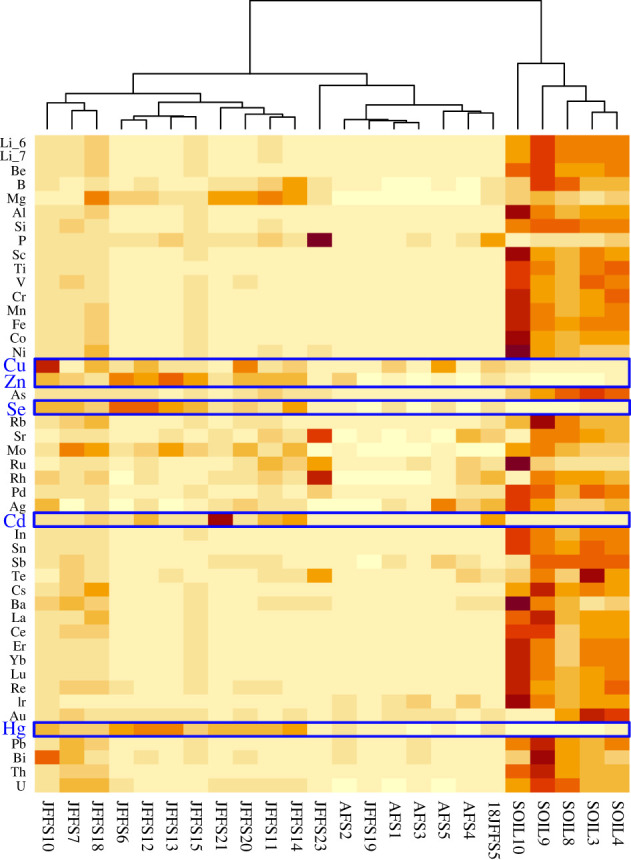
Heatmap showing the results of the two-dimensional hierarchical cluster analysis of faecal and soil samples. Higher colour intensity indicates higher trace element concentrations. Trace elements are on the left, and sample names are on the bottom of the heatmap. Elements showing higher concentrations in faecal samples (Cu, Zn, Se, Cd and Hg) are boxed. The tree above the heatmap indicates the hierarchical clustering of the samples.

The PCA reflected a clear clustering of faecal and soil samples, which explained 65.5% of the total variation in the data. As shown in the biplot in figure 2 and the heatmap in figure 3, five trace elements, copper (Cu), zinc (Zn), selenium (Se), cadmium (Cd) and mercury (Hg) were primarily found in faeces and less so (negligible) in the soil samples.

The heatmap of the two-dimensional hierarchical cluster analysis also revealed three samples (JFFS7, JFFS10, JFFS18) potentially affected by soil contamination. However, we included these samples in further analysis because we only focused on the elements found at low levels in soil but at high levels in the faeces (table 1 for relevant trace element concentration medians and ranges). For the AFS faecal samples, because of the low concentrations of the selected elements present in these samples compared with JFFS, not being able to filter out possible soil contamination for this species does not affect the outcome of this study.

**Table 1.** Trace element concentrations (means and ranges) measured in JFFS and AFS faecal samples in comparison with published levels in other marine species. Only significantly different elements between species are shown. Additional values taken from the literature were also included for perspective. All concentrations are shown based on dry weight. LOQ, limit of quantitation.

species	*n*		Cd (µg g^−1^)	Hg (µg g^−1^)	Zn (µg g^−1^)	Se (µg g^−1^)	source
JFFS	14	median	33.34	0.81	857.47	19.07	this study
*A. philippii philippii*		range	2.90–282.65	0.08–1.20	97.81–1600.17	1.75–38.88	
AFS	5	median	0.59	0.07	155.44	6.21	this study
*A. gazella*		range	0.07–0.70	0.02–0.35	32.77–594.68	3.25–11.25	
sperm whale	2	mean	0.45	1.49	96.92	10.63	Marangi *et al.* [29]
*Physeter macrocephalus*		range	0.41–0.48	1.42–1.56	95.71–98.12	11.01–10.24	
fin whale	2	mean	0.04	<LOQ	52.18	1.06	Marangi *et al.* [29]
*Balaenoptera physalus*		range	0.03–0.04		43.73–60.62	0.84–1.27	
little penguin	6	mean (s.d.)	0.73 (0.44)	0.30 (0.13)	—	5.10 (0.84)	Finger *et al.* [30]
*Eudyptula minor*		range	0.24–1.35	0.18–0.53	—	4.00–6.10	
fish-eating bat	35	mean	—	0.23	—	—	Drinkwater *et al.* [31]
*Myotis vivesi*		range		0.05–0.76			
walrus	16	median	—	0.20	—	—	Rothenberg *et al.* [32]
*Odobenus rosmarus divergens*		range		0.07–0.65			
human adults (Amazon)	17	mean	—	0.05 (0.02)		—	Mendes *et al.* [33]
		range		0.02–0.11			
children (Kasanda, Zambia)	88	median	0.16				Yabe *et al.* [34]
		range	0.07–0.43				

**Figure 4.**
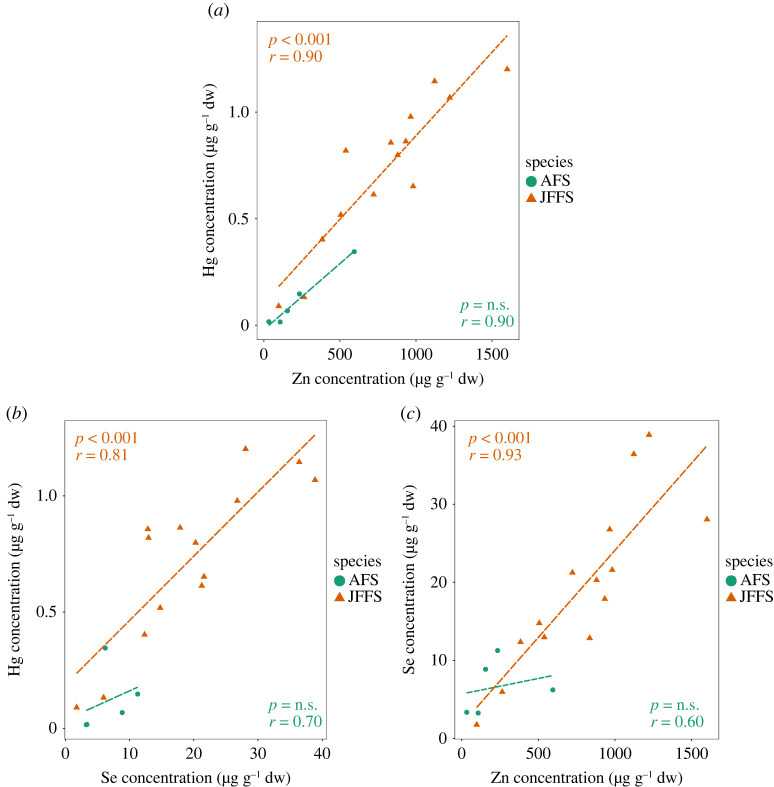
Spearman correlation analysis of elements in faecal samples. (*a*) Correlation between mercury (Hg) and zinc (Zn). (*b*) Correlation between Hg and selenium (Se). (*c*) Correlation between Se and Zn. Data points are coloured according to fur seal species (AFS and JFFS).

From the selected elements identified in pinniped faeces, both Cd and Hg are highly toxic and have no known biological functions. Se, Zn and Cu are essential trace elements, but toxic at higher dosage. Furthermore, Se and Zn are known to contribute in modulating the toxicity of Hg and Cd respectively. Studies looking at heavy metals in animal tissues often describe strong correlations between Hg–Se and Cd–Zn. Here, we investigated if similar patterns were observed in the faeces; which is considered an important excretory route for heavy metals such as Cd and Hg. As expected, Hg was strongly and significantly correlated with Se in the JFFS (figure 4b). This trend was weaker in the AFS but the sample size was also smaller for this species. Interestingly, Zn and Hg concentrations were strongly and significantly correlated in both species (figure 4a). Se and Zn showed a similar pattern as observed between Se and Hg, with a strong and significant pattern only in JFFS (figure 4c). Finally and contrary to our expectations, Cd concentrations only showed a weak and statistically non-significant correlation with Zn. However, Cd was strongly and significantly correlated with Hg in JFFS (*r* = 0.62, *p* = 0.001) (data not shown).

**Figure 5.**
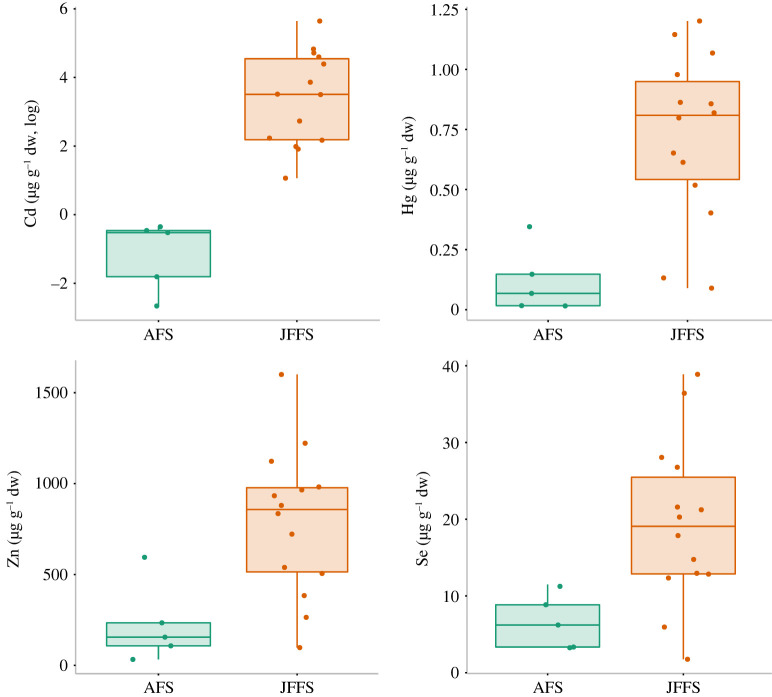
Boxplot showing the concentration of trace elements found in the faecal samples of two fur seal species. Only elements that were significantly different between species are shown (Cd, Hg, Zn and Se).

Next, we investigated differences in faecal concentrations of the different elements between fur seal species. Four of these elements were found to be significantly different between species based on Mann–Whitney non-parametric tests; Cd (W = 0, *p* = 0.01), Hg (W = 4, *p* = 0.01), Se (W = 8, *p* = 0.01) and Zn (W = 8, *p* = 0.01)) (tables 1 and 2, figure 5).

**Table 2.** Summary of the Mann–Whitney tests for the elements found at high levels in fur seal faeces. Each test was carried out with a total of 19 samples (JFFS = 14, AFS = 5). See medians in table 1.

elements	*W*	*p* val^a^		95% CI
Cd	0	<0.001	−6.2	−2.7
Hg	4	0.002	−920	−267.9
Zn	8	0.01	−607.7	−156.8
Se	8	0.01	−23.4	−3.5
Cu	25	n.s.	−203	71.2

^a^Corrected *p* value.

All of these elements were found in significantly higher concentrations in JFFS samples. Levels of Cu did not differ significantly between the species (table 2).

### 3.2. Analysis of prey samples

After looking at the trace elements found in faecal samples, we then focused on levels of these elements in the JFFS diet, specifically Cd and Hg in fish and octopus samples. Myctophid fish and cephalopods such as octopus are described as important components of the JFFS diet [27]. Table 3 summarizes the levels in the prey samples. The octopus hepatopancreas had the highest levels of Hg and Cd of the analysed prey samples (medians = 101.3 ng g^−1^ ww and 76.6 µg g^−1^ ww, *n* = 3, respectively) suggesting this could be the source of Hg and Cd contamination in JFFS.

**Table 3.** Trace element concentrations measured in JFFS diet. Levels in fish and octopus are shown. Other published results of heavy metals in octopus, fish and krill are included for comparison. All concentrations are based on wet weight.

prey	origin	sample type	*n*		Cd (µg g^−1^)	Hg (ng g^−1^)	Zn (µg g^−1^)	Se (µg g^−1^)	P (mg g^−1^)	ref.
myctophids	SPG	whole	11	median	0.26	6.6	10.3	1	6.1	
				range	0.12–0.64	2.0–66.5	8.4–24.4	0.6–2.2	4.5–13.4	
octopus	JFA	hepatopancreas	3	median	76.59	101.3	272.26	4.5	2.09	
*Octopus vulgaris*				range	55.81–133.28	41.5–330.7	248.17–399.11	1.8–5.6	1.90–2.12	
		kidney	3	median	1.52	79	38.78	3.6	2.03	
				range	1.21–12.73	62.1–119.7	22.91–63.25	2–9.3	1.68–2.39	
		gills	3	median	0.09	39.7	19.85	0.8	2.45	
				range	0.04–0.25	18.3–60	18.34–24.58	0.8–1.3	2.10–2.70	
Antarctic krill		whole	4	mean	—	0.02	—	—	—	a
*Euphasia superba*				s.d.		0.01				
Mexican lamp fish^a^	Guaymas Basin	whole	45	median	0.25	—	—	—	—	b
*Triphoturus mexicanus*				range	0.14–0.43					
octopus	Bay of Biscay	whole	13	mean	0.54	**285.52**^a^	—	—	—	c, **d**
*Octopus vulgaris*				s.d.	0.24	**211–422.5**				
octopus^a^	Bay of Biscay	whole	13	mean	—	111.25	—	—	—	d
*Octopus vulgaris*				s.d.		74.25–165.5				
octopus	Adriatic Sea	hepatopancreas	10	mean	7.86	780	—	—	—	e
*Octopus vulgaris*				range	5.47–9.19	370–1380				

^a^Original values were transformed to wet weight asuming 75% of water.

Values in bold came from reference in bold. Ref: (a) Cipro *et al.* [35], (b) Figueiredo *et al.* [36], (c) Bustamante *et al.* [37], (d) Bustamante *et al.* [38], (e) Storelli *et al.* [39].

### 3.3. Analysis of bone samples

The trace element analysis performed on the faeces samples revealed that JFFS were exposed to high levels of Hg and Cd. Analysis of the prey samples (diet), suggested that octopus and possibly other cephalopods are likely to be the primary source of these heavy metals. The next step was to look at the bioavailability of these elements, i.e. the possibility that they are absorbed from the fur seal gut. Bone is a natural store for heavy metals and as bone samples were readily available from dead JFFS seals, we looked at Cd levels in 12 bone samples. These samples included five adult JFFS, five pup JFFS samples, one adult male grey seal (Orkney Islands) and one adult male AFS (South Georgia). The final two samples were included in this analysis only for comparison.

**Figure 6.**
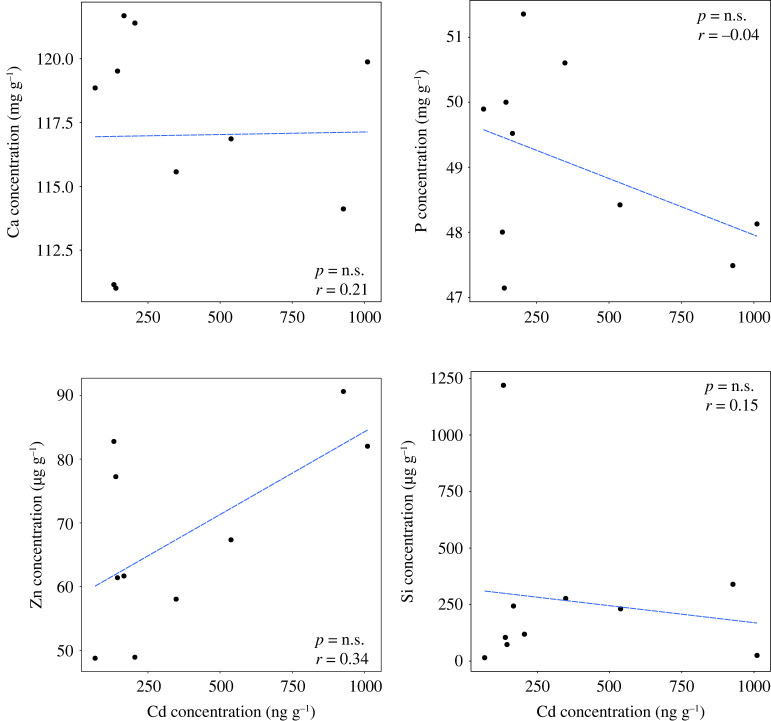
Behaviour of different elements (Ca, P, Zn and Si) found in bone samples in response to cadmium concentrations. Scatter plots show there was only weak or no association between any of the elements and cadmium. The letters *p* and *r* indicate the *p* and *rho* values resulting from the Spearman correlation analysis.

The analyses revealed high concentrations of Cd in JFFS bones (table 4). Due to the known interactions between Cd, Ca, P and Zn in bone [13,43], we investigated for possible correlations between Cd and these essential bone elements. Contrary to our expectations, Cd concentrations did not correlate with any of these elements (figure 6).

**Table 4.** Trace element concentrations (means and ranges) measured in JFFS and AFS bone samples in comparison with published levels in other marine species. Only significantly different elements between species are shown. All concentrations are shown based on dry weight.

age groups	age groups	*n*		Cd (ng g^−1^)	Ca (mg g^−1^)	P (mg g^−1^)	Si (ng g^−1^)	ref.
JFFS	adults	5	median	537.9	119.5	48.4	231.4	
			range	114.5–1010.6	114.1–121.7	47.5–50	25.5–339.9	
	pups	5	median	138.8	115.6	49.9	119.3	
			range	67.1–347.8	111–121.4	47.1–51.4	15.3–1219.6	
	all	10	median	185.9	117.9	489.7	175.4	
			range	67.1–1010.6	111–121.7	471.4–513.6	15.3–1219.6	
grey seal	adult	1	avg replicates	14.6			246.5	
AFS	adult	1	avg replicates	1.2			6.2	
striped dolphin^a^	fetus	13	mean (s.d.)	3 (2)				Honda *et al.* [40]
*Stenella coeruleoalba*	calf (male)	11		40 (30)				
	immature (male)	6		120 (20)				
	mature (male)	5		90 (20)				
	mature (female)	5		180 (50)				
human^a^	adult	37	median	5.75				Lanocha *et al.* [41]
			range	0.25–67.25				
dog^a^		24	median	14.5				Lanocha *et al*. [41]
*Canis familiaris*			range	3.25–52.5				
red fox^a^		12	median	33				Lanocha *et al.* [41]
*Vulpes vulpes*			range	8.5–65				
bottlenose dolphin	Adult	15	mean	50				Lavery *et al*. [42]
*Tursiops aduncus*			range	5–330				

^a^Original values were transformed to wet weight assuming 75% of water.

**Figure 7.**
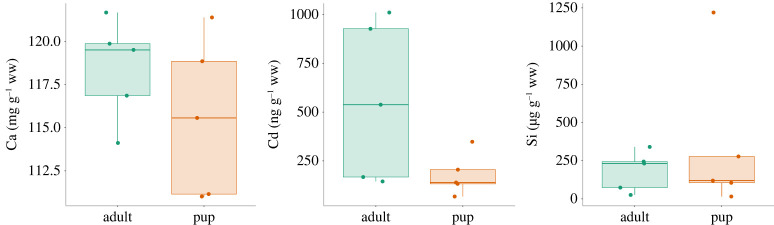
Boxplot showing the concentration of Ca, Cd and Si found in bone samples according to the estimated age group. Age was estimated from bone size.

Finally, we looked at Si, an important sub-trace element in bone known for its role in bone health and often affected by Cd even at minor concentrations. Silicon levels in JFFS bones were high and consistent in both groups (figure 7). However, there was no correlation between Si and Cd. Despite the observed variations, none of the major elements in bone showed statistically significant differences in concentration between age groups (figure 7).

Overall, Cd concentrations were considerably higher in JFFS bones than in GS and AFS bone samples. Silicon concentration was apparently higher in JFFS than in AFS but similar to the value found in the GS bone sample (table 4). However, more samples are required from the AFS and GS to confirm that these differences are statistically significant.

## 4. Discussion

Even though JFFS inhabit a geographically isolated location free of polluting industries and with a low human population density, our study shows that this non-migratory species is exposed to significant levels of contaminats (e.g. heavy metals and microplastics) derived from natural causes as well as human activities. Furthermore, it is likely that in JFFS, foraging behaviour is one of the most critical risk factors of exposure to these. For instance, the most important foraging grounds for JFFS are hundreds of kilometres away from their JFA territory [44]. These hunting areas are located around the plastic-concentrated zone of the South Pacific subtropical gyre [44–46]. Additionally, their most important prey, myctophids and cephalopods, have been associated with contaminant biomagnification and bioaccumulation leading to effects on top predators, such as marine mammal carnivores [47]. A recent study showed that JFFS was the South American pinniped most exposed to microplastic fragments, most likely due to their foraging behaviour [48].

Here, we looked at faecal samples to investigate the exposure of JFFS to heavy metals and compared them with an AFS control group. We also analysed example prey samples to investigate possible sources of these contaminant. Motivated by the results obtained from the faecal samples, we went on to explore Cd concentrations in bone samples. To our knowledge, this is the first study on heavy metal exposure in JFFS in more than two decades [49]. The only available reference on JFFS heavy metal exposure showed that Hg and, to some extent, Cd levels in pup's livers were high compared with previously reported levels in other pinniped [49]. However, liver samples collected from adult AFS inhabiting Bird Island, South Georgia, showed a higher concentration of Cd than the adult JFFS samples included [49]. The AFS study looked at 11 adult females over 5 years old, in comparison two male samples were included in the JFFS study, a subadult (under 3 years old) and an adult of undetermined age [49,50]. As discussed later, age and sex are relevant when comparing heavy metal accumulation.

Additionally, and despite our modest sample size of individuals, this is the first report on heavy metals in octopus (*Octopus vulgaris*), a species that is of great relevance as both a local human food source and also to the local economy. Although we only focused on the non-edible tissues.

### 4.1. Findings in faecal samples

After controlling for potential soil contamination, only five elements were strongly associated with the faecal samples, including the heavy metals Cd and Hg. Apart from Cu, the other four trace elements (Cd, Hg, Zn, Se) were significantly higher in JFFS compared with AFS faeces.

In marine mammals, diet is one of the most important sources of exposure of heavy metals and other pollutants [29,51,52]. Thus, the trophic level at which marine species feed will largely influence the levels of exposure to contaminants. We believe that prey selection is likely to explain the differences observed between JFFS and AFS faecal samples. For instance, the levels of Cd, Hg, Zn and Se found in sperm whale (*Physeter macrocephalus*) faecal samples were higher than those found in its sympatric species, the Mediterranean fin whale (*Balaenoptera physalus*) (table 1) [29]. Interestingly, AFS and fin whales feed predominantly on krill (*Euphausia superba* and *Meganyctiphanes norvegica,* respectively), whereas sperm whales and JFFS prey mostly on cephalopods and fish [27,44,53–55].

The levels of Hg in the JFFS faecal samples analysed here were also higher than many of the previously reported concentrations in other mammalian species faecal samples (table 1) [29–33]. From the literature, only sperm whale faecal samples showed higher concentrations of Hg [29]. To give some perspective, Hg in JFFS was 12 times higher than AFS, four times higher than walruses and 16 times higher than the levels found in adult human faeces from a small Amazonian community whose diets rely largely on mercury-contaminated fish [32,33].

Animals have mechanisms to eliminate and detoxify toxic metals after exposures. A study looking at Hg elimination in the fur and faeces of captive Baltic GS (*Halichoerus grypus grypus*) found that faeces were an efficient elimination route for total Hg [56]. They estimated that faeces accounted for up to 48% of Hg elimination. A similar rate was identified in bottlenose dolphins [57]. Meanwhile, in this study, there was a strong correlation between Hg and Se concentrations in JFFS but not AFS faeces. Accumulating evidence strongly suggests Se plays an essential role in the detoxification process of methylmercury, the most toxic form of this heavy metal, as well as inorganic Hg (iHg) [58,59]. Furthermore, the formation of inert HgSe particles has been suggested as a critical Hg-detoxification strategy in marine animals, where either the particles themselves or positive correlations between Hg and Se have been documented in various internal tissues, fur and feathers [56,60–65]. Thus, the correlation between Hg and Se found here may reflect the formation of HgSe particles as part of the detoxification mechanism in JFFS.

A particularly compelling finding of the faecal samples analysis was the surprisingly high levels of Cd in JFFS faeces. When comparing the median concentrations between species, this value was 57 times higher than that found in AFS samples. Even the minimum value in JFFS faecal samples was higher than any of the maximum values reported for other mammalian species (table 1). Furthermore, JFFS faecal Cd levels were more than 200 times higher than those found in the faeces collected from children inhabiting a Cd-polluted mining town in Zambia [34]. Despite the lower Cd levels detected in AFS faecal samples compared with JFFS samples, these were not necessarily ‘low’ for mammals *per se*. The median value for this species was three times higher than the concentrations observed in the Zambian children previously mentioned and 14 times higher than the levels found in fin whales (table 1) [29,34].

### 4.2. Prey samples

In marine mammals, heavy metal exposure will most likely depend on the trophic level at which individuals feed [14,29,51,61,66,67]. However, heavy metal exposure is also linked to specific environmental characteristics. For instance, a clear link has been shown between ocean depth and heavy metal contamination in different trophic webs [68,69]. Furthermore, [70] showed that Hg accumulation in birds feeding on mesopelagic (below 200 m sea depth) squid and fish was higher than when they fed on epipelagic fish and squid (above 200 m sea depth) [70].

The limited information on the JFFS diet suggests this species has a mesopelagic ichthyo-tentophagous diet, which means their diet is mainly based on mesopelagic fish, mainly myctophids, and squid [27]. Additionally, the local community has reported that octopus inhabiting the coastal benthic floor of the archipelago forms part of the JFFS diet. Based on sample availability, we analysed heavy metal concentrations in two different types of prey known to be consumed by JFFS, *Symbolophorus sp*, a mesopelagic myctophid fish, and *Octopus vulgaris*, a benthic cephalopod.

As expected, the largest concentrations of Cd were found in octopus samples. As previously reported in the literature, the hepatopancreas, also known as the digestive gland, was the organ with the highest Cd concentration [37,71,72]. Furthermore, the Cd concentrations in the octopus hepatopancreas collected from Robinson Crusoe Island was much higher compared with other studies, but Hg concentrations were relatively low [37–39,73]. To understand better how different preys contribute to heavy metal exposure, future work would need to include other cephalopod species such *Onychoteuthis banskii* (now reclassified as *Onychoteuthis aequimanus*), which were the most abundant squid beaks found in JFFS scats, especially in females [27]. Due to their mesopelagic foraging behaviour, we hypothesize that these Cd levels would be even higher than in *O. vulgaris*.

When looking at Cd and Hg levels in myctophid samples, levels were low compared with those found in octopus organs. However, this difference is likely to be inflated because whole fish were compared with individual organs in octopuses. Unfortunately, due to the size and fragility of the fish samples after defrosting, it was not possible to separate specific organs to allow a fair comparison. A recent study on trace elements in myctophids from the Gulf of California showed similar levels to those presented here. These levels were among the highest compared with myctophids from other regions [36]. Furthermore, Cd levels measured in whole individuals from various octopus species such as *O. vulgaris* (Bay of Biscay) and *Eledone cirrhosa* (Faroe Island), were only 1.8 and 10 times, higher than those observed in the myctophid samples from this study [37]. In comparison, there was an almost 300-fold difference between the myctophids and octopus hepatopancreas analysed here. Thus, although cephalopods are likely to be the main Cd source, myctophids could still be contributing to Cd exposure in JFFS. On the other hand, as with octopus, Hg levels in myctophid samples analysed here compared with other studies were not particularly high [35]. Thus, the source of Hg in JFFS remains unclear.

Regarding the AFS diet, it is known that this species feeds predominantly on krill. However, the composition of their diets may vary depending on environmental characteristics and differences in foraging behaviour between colonies. For instance, when water temperature increases, krill populations decline and AFS may then shift their prey selection toward cephalopods [74]. Other researchers have also shown an association between prey preference and different AFS genetic lineages. For example, some colonies, such as the one included in this study, feed almost exclusively on krill, while others prey primarily on fish and practically no krill [75]. Thus, the low Hg and Cd levels found in AFS faecal samples in this study nicely reflect the colony preference for krill. This intra-species diet difference makes AFS an attractive target to study the effects of differences in heavy metal exposure on fur seals.

### 4.3. Bone samples

Based on the high levels of Cd in the faeces, it was logical to assume high levels of this heavy metal in bones, an important site for its accumulation. The ICP-MS data from the bone samples analysis confirmed the hypothesis that Cd was indeed bioavailable from the gut and accumulated in the bones. JFFS bone samples showed high levels of Cd compared with other marine and terrestrial mammalian species [40–42]. Cd is known for its detrimental effects on bone mineralization [13,43]. For instance, high levels of this heavy metal negatively affect Zn and Ca concentrations in bones even at very low concentrations [13,76–78]. However, despite the high bone Cd concentrations, there was no influence on the composition of these essential elements. These results suggests JFFS bones are resilient to Cd accumulation and toxicity.

Previous studies have shown that marine mammals exposed to high levels of heavy metals have developed mechanisms to tolerate such high levels of contamination. Most of these studies link heavy metal tolerance to increased expression of metallothioneins and show the important role of the liver and kidneys in the detoxification and excretion process [15,79,80]. To our knowledge, this is the first time bone resilience to high levels of Cd exposure has been reported in these species.

Over the past 20 years, silicon (Si) has been reported to be associated with bone health [81]. Although the exact role Si plays in bone is not established, evidence suggests that Si may be actively involved in the bone mineralization process [82–84]. Furthermore, positive associations between increased silicon intake and higher bone mineral density have been reported [83,85–87]. Bone Si levels in JFFS were also analysed in this study. Unfortunately, there are no other reports on Si levels in marine mammals to compare our values with. For this reason, the cranial bones from one adult male AFS and one adult male grey seal were analysed for Si.

Interestingly, the AFS bone samples had minimal levels of Cd and Si compared with the JFFS and grey seal bone samples. The grey seal bone sample, on the other hand, had similar Si levels to the median values from JFFS bones but much lower Cd concentrations. Similar to JFFS, GS feed at a relatively high trophic level, taking a wide variety of prey, including fish, cephalopods and crustaceans. Additionally, foraging ecology in GS differs significantly between males and females [88]. This difference has also been shown to influence heavy metal exposure in this species. Females have at least four times higher Cd levels compared with males in the kidney, liver and muscle [61]. From the above, we hypothesize that high levels of Si may be a general trait of species that feed at higher trophic levels regardless of any variation in feeding behaviour within the species. Future work should include more samples, look at species with different trophic ecologies and determine the sex and age of the samples.

We did not investigate the bioavailability of Hg in the JFFS by analysing Hg in relevant tissue samples e.g. liver, fat tissue, kidneys. Furthermore, prey samples analysed in this study did not fully explain the source of this contamination. Nevertheless, we would expect high concentrations of this metal in key organs such as the liver, kidneys and muscles of JFFS. In addition, we would also expect to find high levels of metallothioneins, HgSe crystals and most of the methylmercury being stored in less sensitive cells such as muscle.

Finally, the prey samples included in this study did provide clues as to the source of this heavy metal contamination. Identifying the source of Hg contamination could provide critical information to the local community. Diet is an important route of exposure for methylmercury. Although marine mammals show some resistance, humans, especially those eating large quantities of contaminated seafood, are particularly vulnerable to the negative effects. In the context of the JFA, the findings in this study might be of great relevance regarding to food security. Carnivorous fish, octopus and lobster are important parts of the local diet, but to the best of our knowledge, there have not been any studies on heavy metal contamination in the human food chain.

## 5. Conclusion and future work

This study investigated heavy metal exposure in the JFFS. Although cephalopod-rich diets play an important role in the transfer of Cd to top predators, our study shows that JFFS are exposed to and accumulate high levels of this toxic metal compared with levels reported for marine mammals with a similar diet. Our lack of knowledge on heavy metal contamination in other non-octopus cephalopods makes it challenging to identify how different prey species influence the Cd levels observed in JFFS faeces. Additionally, a recent study showed that JFFS was highly exposed to plastic debris [48], which may enhance their exposure to Cd. Further studies using Cd isotope fractionation in JFFS faeces to look at the proportion of Cd from natural versus human sources will contribute to our understanding of the trophic transfer of human-originated contaminants in remote locations.

In general, marine mammals have developed efficient strategies to counteract the high levels of heavy metals to which they are naturally exposed. Our understanding of heavy metal tolerance, including Cd, is based on the study of key organs such as the liver and kidneys. Despite the known toxic effects of Cd in terrestrial mammal bones, little is known about the effects of this heavy metal in marine mammals. Here, we identified bones as a novel target to study resistance adaptations to heavy metals in marine mammals. Furthermore, to our knowledge, this is the first study reporting Si concentrations in marine mammal bones. We believe the high level of Si observed in JFFS bones may be associated with the absence of mineral changes seen with Cd exposure in land mammals. Understanding the possible role Si plays in protecting bones from the Cd damage would be a significant contribution to biomedical research.

Additionally, our study highlights the need to monitor the risk of heavy metal contamination in human communities inhabiting oceanic islands, despite the degree of isolation and pristinity. Our findings on heavy metal contamination in JFFS support our recommendation to assess further the risk of consuming contaminated seafood by the JFA local community. Identifying fishery products naturally carrying high levels of heavy metals and understanding how these metals are distributed within those organisms will allow the community to make informed food choices (e.g avoiding certain internal organs such as the hepatopancreas of natural heavy metal accumulators). This information is particularly relevant during pregnancy and early fetal development.

## Data Availability

Data and relevant code for this research work are stored in GitHub: https://github.com/Cotissima/JFFS_HeavyMetal_analysis and have been archived within the Zenodo repository: https://zenodo.org/badge/latestdoi/488014527.
